# Endothelial Mitochondria Transfer to Melanoma Induces M2-Type Macrophage Polarization and Promotes Tumor Growth by the Nrf2/HO-1-Mediated Pathway

**DOI:** 10.3390/ijms25031857

**Published:** 2024-02-03

**Authors:** Fu-Chen Kuo, Hsin-Yi Tsai, Bi-Ling Cheng, Kuen-Jang Tsai, Ping-Chen Chen, Yaw-Bin Huang, Chung-Jung Liu, Deng-Chyang Wu, Meng-Chieh Wu, Bin Huang, Ming-Wei Lin

**Affiliations:** 1 School of Medicine, College of Medicine, I-Shou University, Kaohsiung 82445, Taiwan; ed100418@edah.org.tw; 2 Department of Obstetrics & Gynecology, E-Da Hospital, I-Shou University, Kaohsiung 82445, Taiwan; 3 Department of Medical Research, E-Da Hospital and E-Da Cancer Hospital, I-Shou University, Kaohsiung 82445, Taiwan; y7952pipi@gmail.com; 4 School of Pharmacy, Kaohsiung Medical University, Kaohsiung 80708, Taiwan; yabihu@kmu.edu.tw; 5 Department of Biological Sciences, National Sun Yat-sen University, Kaohsiung 80424, Taiwan; biling128@gmail.com (B.-L.C.); lajajaf@gmail.com (P.-C.C.); 6 Department of General Surgery, E-Da Cancer Hospital, I-Shou University, Kaohsiung 82445, Taiwan; ed108937@edah.org.tw; 7 Regenerative Medicine and Cell Therapy Research Center, Kaohsiung Medical University, Kaohsiung 80708, Taiwan; pinkporkkimo@yahoo.com.tw (C.-J.L.); dechwu555@gmail.com (D.-C.W.); 8 Department of Internal Medicine, Kaohsiung Medical University Hospital, Kaohsiung 80708, Taiwan; 930293@mail.kmuh.org.tw; 9 Department of Internal Medicine, Kaohsiung Municipal Ta-Tung Hospital, Kaohsiung 80145, Taiwan; 10 Department of Biomedical Science and Environmental Biology, College of Life Science, Kaohsiung Medical University, Kaohsiung 80708, Taiwan; 11 Department of Medical Research, Kaohsiung Medical University Hospital, Kaohsiung 80708, Taiwan; 12 Department of Nursing, College of Medicine, I-Shou University, Kaohsiung 82445, Taiwan

**Keywords:** melanoma, endothelial cells, mitochondrial transplantation, Nrf2, tumor microenvironment, M2-type macrophage, tumor growth

## Abstract

Gynecologic tract melanoma is a malignant tumor with poor prognosis. Because of the low survival rate and the lack of a standard treatment protocol related to this condition, the investigation of the mechanisms underlying melanoma progression is crucial to achieve advancements in the relevant gynecological surgery and treatment. Mitochondrial transfer between adjacent cells in the tumor microenvironment regulates tumor progression. This study investigated the effects of endothelial mitochondria on the growth of melanoma cells and the activation of specific signal transduction pathways following mitochondrial transplantation. Mitochondria were isolated from endothelial cells (ECs) and transplanted into B16F10 melanoma cells, resulting in the upregulation of proteins associated with tumor growth. Furthermore, enhanced antioxidation and mitochondrial homeostasis mediated by the Sirt1-PGC-1α-Nrf2-HO-1 pathway were observed, along with the inhibition of apoptotic protein caspase-3. Finally, the transplantation of endothelial mitochondria into B16F10 cells promoted tumor growth and increased M2-type macrophages through Nrf2/HO-1-mediated pathways in a xenograft animal model. In summary, the introduction of exogenous mitochondria from ECs into melanoma cells promoted tumor growth, indicating the role of mitochondrial transfer by stromal cells in modulating a tumor’s phenotype. These results provide valuable insights into the role of mitochondrial transfer and provide potential targets for gynecological melanoma treatment.

## 1. Introduction

Melanoma, the most aggressive type of skin cancer, grows rapidly and can metastasize to other organs. In particular, gynecologic tract melanoma has a poor prognosis, with a 5-year survival rate lower than 25%. Because of the low survival rate and the lack of a standard treatment approach related to gynecologic tract melanoma [1,2,3], elucidation of the mechanisms underlying melanoma progression is crucial for achieving advancements in relevant gynecological surgery and treatment. Studies have indicated that extracellular signal-regulated kinase (ERK) and phosphoinositide 3-kinase/protein kinase B (AKT) pathways play major roles in the progression of melanoma [4,5,6]. In addition, genes implicated in cell cycle progression, including cyclin D1 and cyclin E, are commonly amplified in melanoma [7]. Furthermore, the progression of melanoma involves interactions with surrounding stromal cells in the tumor microenvironment (TME). Interactions between melanoma cells and endothelial cells (ECs) are crucial in tumor biology and enable tumor cells to undergo proliferation and metastasis [8]. In addition, such interactions—which occur through paracrine communication, direct contact, or gap junctions—facilitate the release of various signaling molecules—including growth factors, extracellular vesicles, and mitochondria—into the TME [8,9]. In the TME, M1-type antitumor macrophages expressing inducible nitric oxide synthase (iNOS) are polarized into M2-type macrophages expressing arginase 1 (Arg1), thereby promoting cancer cell proliferation and metastasis. Furthermore, cancer cells stimulate the differentiation of nonactivated macrophages into an M2-like tumor-associated macrophage (TAM) phenotype through the action of transforming growth factor β (TGF-β) [10,11,12,13].

Mitochondria, also regarded as endosymbiotic organelles, not only produce the majority of cellular energy, but also are involved in cellular exchanges, modulating the fate and function of cells [14,15]. Nuclear respiratory factor 2 (Nrf2) is a key modulator of peroxisome proliferator-activated receptor-gamma coactivator 1-α (PGC-1α)-mediated mitochondrial activity and protects against reactive oxygen species (ROS) generation and oxidative damage [16,17]. Mitochondrial transfer between adjacent cells occurs through tunneling nanotubes, microvesicles, gap junctional intercellular communication, and extrusion [9,15] the transfer of mitochondria from vascular smooth muscle cells to mesenchymal stem cells (MSCs) enhances cell proliferation [18]. In addition, the mitochondria from MSCs alleviate stress in patients with osteoarthritis [19]. Furthermore, the mitochondria obtained from bone-marrow-derived MSCs can rescue cardiomyocytes from ischemia-induced oxidative stress and cell death [20]. MSCs donate their healthy mitochondria to damaged cells, thereby enhancing the recipient cells’ oxidative stress resistance, proliferation, and antiapoptotic capability [21]. In addition, such intercellular mitochondrial transfer is involved in the regulation of cancer progression [21,22,23,24]. Nevertheless, the mechanisms through which endothelial mitochondria affect melanoma progression, and subsequently regulate TAMs to promote tumor growth after their transfer, remain to be elucidated.

In recent years, mitochondrial transplants have emerged as a method for examining the functions of recipient cancer cells after their uptake of mitochondria from donor cells in the TME [25,26]. A common approach to mitochondrial transplants involves co-culturing recipient cells with isolated mitochondria from donor cells [27,28]. In the present study, we transplanted mitochondria from ECs into melanoma cells and used signaling pathways and tumor xenograft animal models to investigate the role of mitochondrial transfer in melanoma progression.

## 2. Results

### 2.1. Transplanted Endothelial Mitochondria Upregulated Mitochondrial Biogenesis with Mediation by the Redox-Sensitive Transcription Factor Nrf2

**Figure 1:**
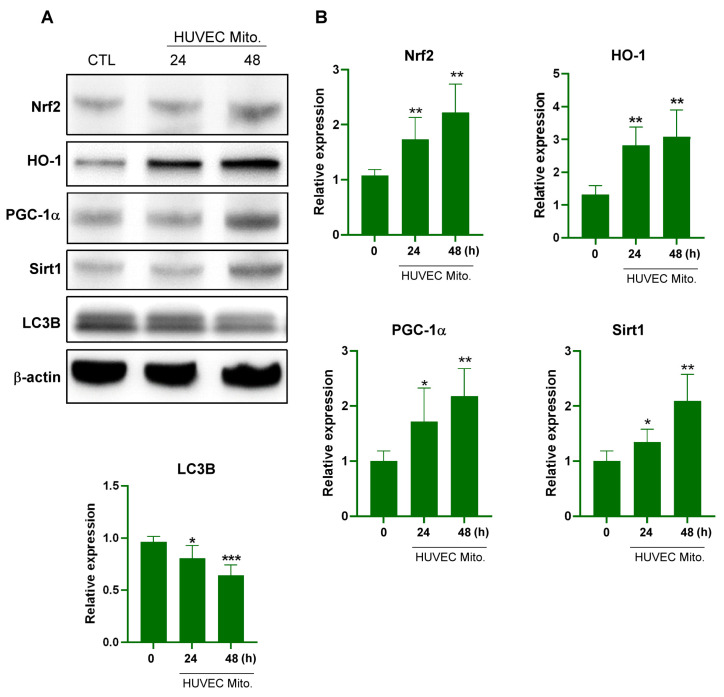
Transplanted HUVEC mitochondria upregulated the expression of antioxidant and mitochondria biogenesis proteins in B16F10 cells. (**A**) Western blot analysis demonstrating the protein expression levels of Nrf2, HO-1, PGC-1α, Sirt1, and LC3B in B16F10 cells with or without HUVEC mitochondrial transplantation at 24 and 48 h. (**B**) Quantitative analysis of Nrf2, HO-1, PGC-1α, Sirt1, and LC3B expression levels in B16F10 cells after the transplantation of HUVEC mitochondria, as determined through Western blotting at 24 and 48 h. Data are presented as the mean ± standard error after ≥3 independent experiments. Statistical significance was assessed using Student’s *t* test: * *p* < 0.05, ** *p* < 0.01, *** *p* < 0.005.

Silent mating-type information regulation 2 homolog (Sirt1), an NAD^+^-dependent class III histone deacetylase, plays a key role in both mitochondrial biogenesis and cellular redox homeostasis through the PGC-1α and Nrf2 pathways [29]. Nrf2, a redox-sensitive transcription factor, subsequently induces heme oxygenase-1 (HO-1) expression. Upregulation of HO-1 is vital for protecting cancer cells against oxidative stress [30]. To investigate the intracellular effects of endothelial mitochondrial transplantation, mitochondria isolated from human umbilical vein ECs (HUVECs; 5 × 10^6^ cells) were incubated with 5 × 10^6^ B16F10 melanoma cells. After 24 or 48 h of coincubation, we examined the expression levels of Nrf2, HO-1, PGC-1α, Sirt1, and autophagic biomarker LC3B. The results indicated time-dependent increases in the levels of Nrf2, HO-1, PGC-1α, and Sirt1 proteins and a reduction in the level of LC3B (Figure 1).

### 2.2. Transplanted Endothelial Mitochondria Enhanced Melanoma Cells’ Viability through Activation of ERK and AKT Signaling and Suppression of Apoptosis

**Figure 2:**
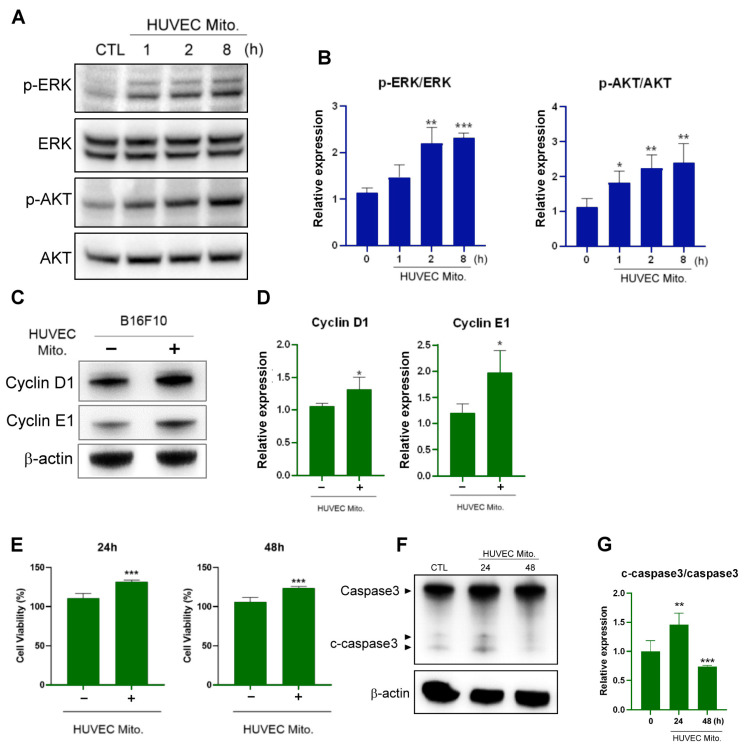
Transplanted HUVEC mitochondria-activated proliferative signaling and inhibited apoptosis in B16F10 cells. (**A**) Western blot analysis demonstrating the activation of the ERK and AKT signaling pathways at 1, 2, and 8 h following HUVEC mitochondrial transplantation. (**B**) Quantitative analysis of the phosphorylation ratios of ERK and AKT after treatment with HUVEC mitochondria, as determined through Western blotting. (**C**) Expression levels of cyclin D1 and cyclin E in B16F10 cells treated with HUVEC mitochondria for 48 h, as determined through Western blotting. (**D**) Quantitative analysis of cyclin D1 expression and cyclin E expression. (**E**) Viability of B16F10 cells at 24 and 48 h after treatment, as evaluated using the CCK8 assay. (**F**) Caspase-3 and cleaved caspase-3 levels in B16F10 cells, analyzed through Western blotting at 24 and 48 h after treatment with HUVEC mitochondria. (**G**) Quantification of the cleaved caspase-3-caspase-3 expression ratio. Data are presented as the mean ± standard error of the mean after ≥3 independent experiments. Statistical significance was assessed using a two-tailed Student’s *t*-test: * *p* < 0.05, ** *p* < 0.01, *** *p* < 0.005.

After confirming that HUVEC mitochondria can be transplanted into B16F10 cells, a process that affects both mitochondrial biogenesis and cellular redox homeostasis, we identified the proteins involved in cancer cell proliferation and apoptosis. The results revealed that the transplantation of HUVEC endothelial mitochondria significantly enhanced the phosphorylation capabilities of ERK and AKT in a time-dependent manner (Figure 2A,B). The expression of cyclin D1 and cyclin E, which regulate cell proliferation, is deregulated in many cancers, including melanoma [31]. We observed upregulated expression of both cyclin D1 and cyclin E (Figure 2C,D). Moreover, the transplantation of endothelial mitochondria enhanced the viability of B16F10 cells (Figure 2E). The cleaved caspase-3 was increased at 24 h and then reduced the level of apoptotic protein cleaved caspase-3 at 48 h (Figure 2F,G).

### 2.3. Transplanted Endothelial Mitochondria-Suppressed AKT, Oxidative Stress and Apoptosis of Melanoma Cells through Activation of Nrf2-Mediated Signaling

**Figure 3:**
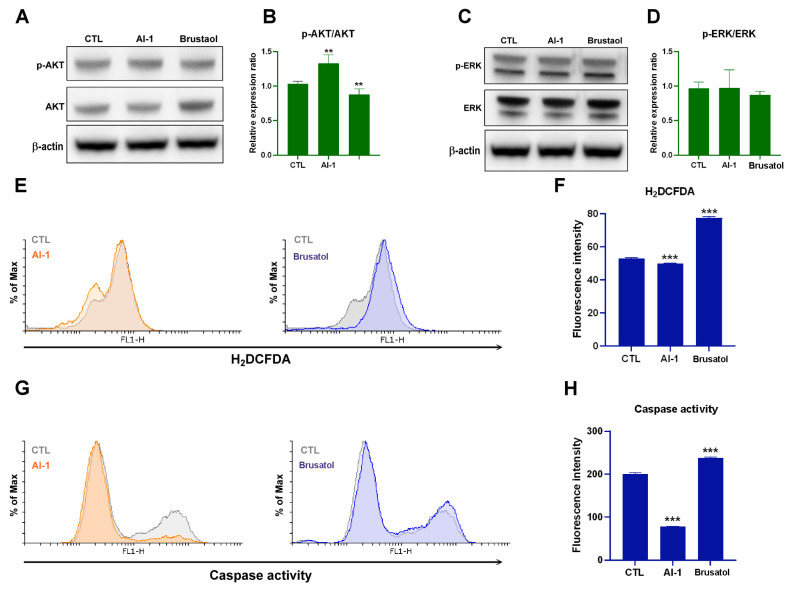
Effects of AI-1 and brusatol on B16F10 Cells. (**A**) Western blot analysis comparing the ratio of p-AKT/AKT in B16F10 cells treated with AI-1 (10 μ) and brusatol (40 nM) at 48 h. (**B**) Quantitative analysis of the p-AKT/AKT ratio after treatment with AI-1 and brusatol, as determined through Western blotting at 48 h. (**C**) Western blot analysis comparing the ratio of p-ERK/ERK in B16F10 cells treated with AI-1 (10 μ) and brusatol (40 nM) at 48 h. (**D**) Quantitative analysis of the p-ERK/ERK ratio after treatment with AI-1 and brusatol, as measured through Western blotting at 48 h. (**E**) Cellular ROS levels of B16F10 cells treated with AI-1 (10 μ) and brusatol (40 nM) at 48 h, as determined through flow cytometry. (**F**) Quantification of ROS levels in B16F10 cells treated with AI-1 (10 µM) and brusatol (40 nM) at 48 h, as determined through flow cytometry. (**G**) Evaluation of caspase activity in B16F10 cells treated with AI-1 (10 µM) and brusatol (40 nM) at 48 h, as determined through flow cytometry. (**H**) Quantitative analysis of caspase activity in B16F10 cells treated with AI-1 (10 µ) and brusatol (40 nM) at 48 h, as determined through flow cytometry. Data are presented as the mean ± standard error of the mean after ≥3 independent experiments. Statistical significance was assessed using a two-tailed Student’s *t*-test: ** *p* < 0.01, *** *p* < 0.005.

To determine whether Nrf2 plays a pivotal role in AKT or ERK signaling, oxidative stress adaption, or apoptosis in melanoma cells after the uptake of endothelial mitochondria, we used brusatol, an Nrf2 inhibitor, and AI-1, an Nrf2 activator, to evaluate the activities of AKT, ERK, ROS, and apoptotic protein caspase-3. As shown in Figure 3, treatment with brusatol (40 nM) for 48 h revealed that the suppression of Nrf2 inhibited AKT signaling, increased ROS, and promoted apoptosis. By contrast, AI-1-activated AKT signaling reduced ROS and inhibited apoptosis, specifically AI-1- or brusatol-regulated AKT phosphorylation but not ERK (Figure 3).

### 2.4. Transplantation of Endothelial Mitochondria-Promoted Melanoma Tumor Growth through Nrf2-Mediated Pathway in a Tumor Xenograft Animal Model

**Figure 4:**
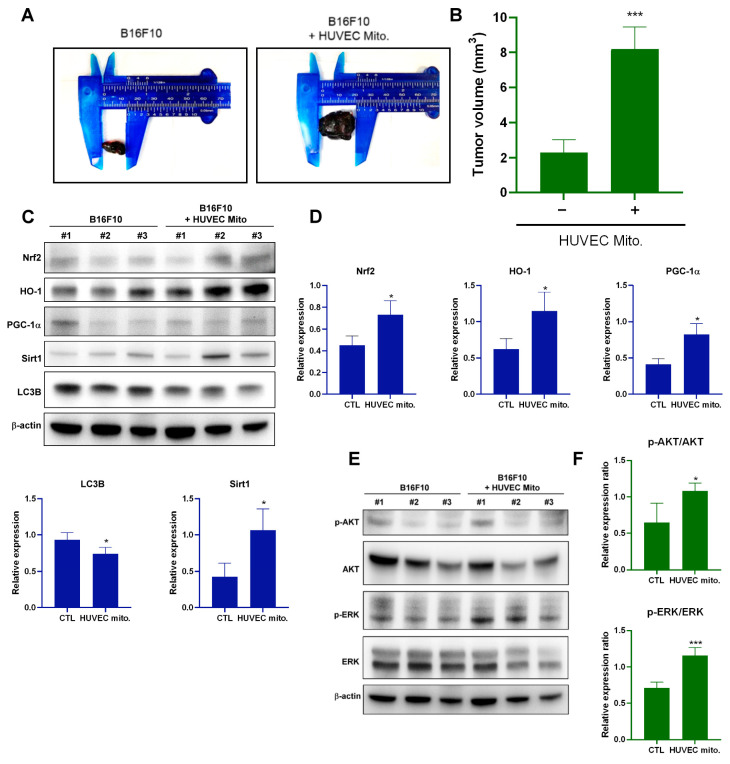
Xenograft analysis of melanoma tumor growth. (**A**) Mice were inoculated with B16F10 cells that underwent HUVEC mitochondrial transplantation and were monitored for 10 days. (**B**) Tumor size analysis in six mice. (**C**) Western blot analysis comparing the protein expression levels of Nrf2, HO-1, PGC-1α, Sirt1, and LC3B in B16F10 tumors with or without HUVEC mitochondrial transplantation at 10 days. (**D**) Quantification of the protein expression levels of Nrf2, HO-1, PGC-1α, Sirt1, and LC3B in B16F10 tumors with or without HUVEC mitochondrial transplantation at 10 days. (**E**) Western blot analysis of AKT or ERK signaling in B16F10 tumors with or without HUVEC mitochondrial transplantation at 10 days. (**F**) Quantitative analysis of p-AKT/AKT and p-ERK/ERK ratios in B16F10 tumors with or without HUVEC mitochondrial transplantation at 10 days. Data are presented as the mean ± standard error of the mean after ≥3 independent experiments. Statistical significance was assessed using a two-tailed Student’s *t*-test: * *p* < 0.05, *** *p* < 0.005.

Mice were inoculated with B16F10 cells containing HUVEC mitochondria. After 10 days, the tumors in these mice were larger than those in the mice injected with B16F10 cells without HUVEC mitochondria (Figure 4A,B). Protein expression in the tumors in these mice was similar to that observed in melanoma cells after the uptake of endothelial mitochondria (Figure 4C–F). These results indicate that the uptake of endothelial mitochondria by melanoma cells promotes tumor growth through AKT/ERK and Nrf2-mediated pathways.

### 2.5. Transplant of Endothelial Mitochondria to Melanoma Upregulated Matrix Metallopeptidase 9, TGF-β1 and Induced M2-Type TAM in a Tumor Xenograft Animal Model

**Figure 5:**
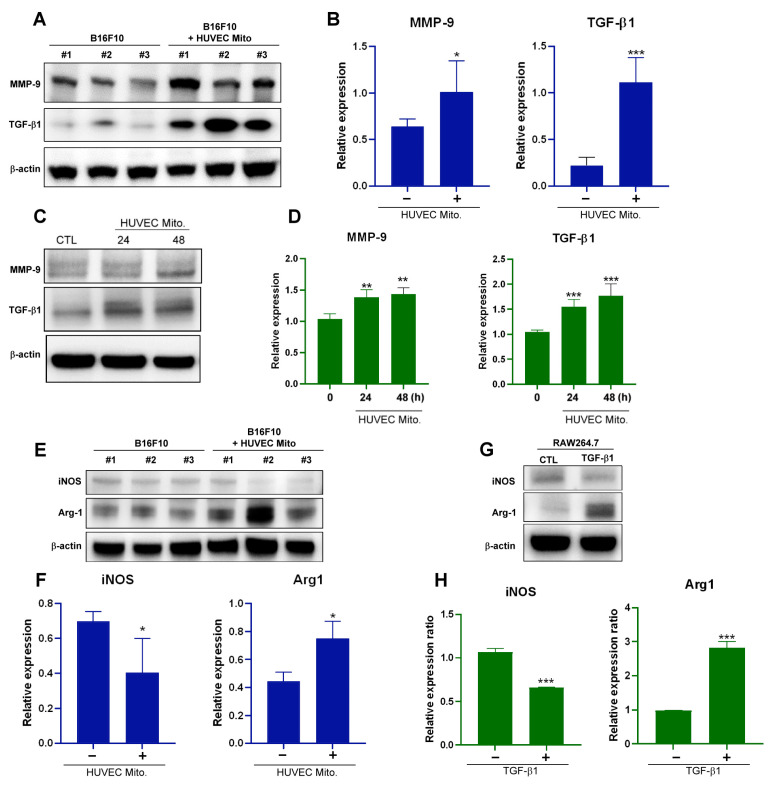
Xenograft analysis of melanoma tumor growth. (**A**) Western blot analysis comparing the protein expression levels of MMP-9 and TGF-β1 in B16F10 tumors with or without HUVEC mitochondrial transplantation at 10 days. (**B**) Quantitative analysis of MMP-9 and TGF-β1 protein expression levels in B16F10 tumors with or without HUVEC mitochondrial transplantation at 10 days. (**C**) Western blot analysis comparing the protein expression levels of MMP-9 and TGF-β1 in B16F10 cells with and without HUVEC mitochondrial transplantation at 24 or 48 h. (**D**) Quantification of MMP-9 and TGF-β1 expression after treatment with HUVEC mitochondria, as measured through Western blotting at 24 or 48 h. (**E**) Western blot analysis comparing iNOS and Arg1 protein levels in B16F10 tumors with or without HUVEC mitochondrial transplantation at 10 days. (**F**) Quantitative analysis of iNOS and MMP-9 protein levels in B16F10 tumors with or without HUVEC mitochondrial transplantation at 10 days. (**G**) Western blot analysis comparing iNOS and Arg1 protein expression levels in RAW264.7 cells treated with or without TGF-β1 (20 ng/mL) at 48 h. (**H**) Quantification of iNOS and MMP-9 protein levels in RAW264.7 cells treated with or without TGF-β1 (20 ng/mL) at 48 h. Data are presented as the mean ± standard error of the mean after ≥3 independent experiments. Statistical significance was assessed using a two-tailed Student’s *t*-test: * *p* < 0.05, ** *p* < 0.01, *** *p* < 0.005.

Nrf2 activates several oncogenes, including matrix metallopeptidase 9 (MMP9). MMP9 is the main enzyme able to remodel the extracellular matrix by favoring the tumor invasive processes [32,33]. Protein expression in the tumors and melanoma cells revealed the upregulation of MMP-9 and TGF-β1 after the transplantation of endothelial mitochondria (Figure 5A–D). The TGF-β signaling pathway plays a role in melanoma metastasis and macrophage polarization. TGF-β induces macrophage polarization into M2-like TAMs. These cells expressed the enzyme Arg1 display tumorigenic functions with increased metastatic potential and tumor cell proliferation [10,11,12,13,34]. TGF-β induces macrophages to express the M2-type marker Arg1 and downregulates the expression of the M1-type marker iNOS. In the mitochondrial transplantation groups, Arg1 was upregulated not only in a tumor xenograft animal model, but also in a B16F10 melanoma cell model. However, iNOS was downregulated in the mitochondrial transplant groups but not in the control groups (Figure 5E,F). Finally, TGF-β-induced macrophage Arg1 expression was confirmed in TGF-β1-treated RAW264.7 macrophage cells (Figure 5G,H).

### 2.6. Transplant of Endothelial Mitochondria into Melanoma Cells Upregulated TGF-β Expression through the Nrf2-Mediated Pathway

**Figure 6:**
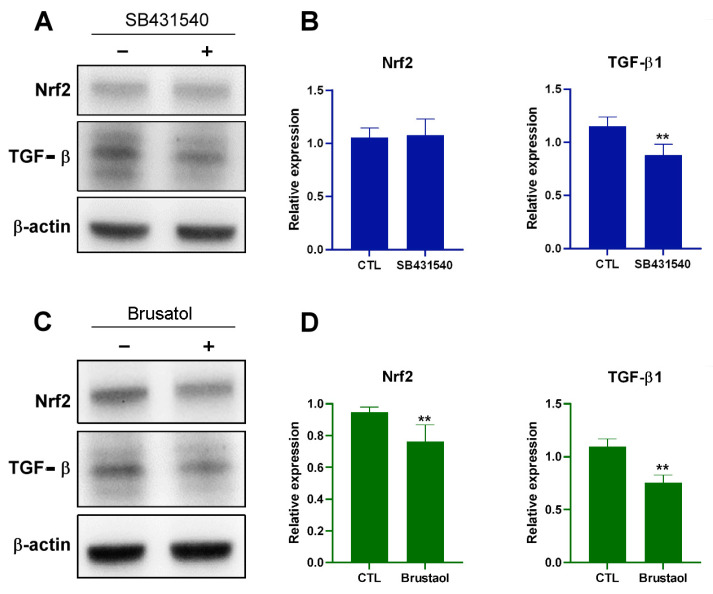
Effects of SB431540 and brusatol on B16F10 Cells. (**A**) Western blot analysis comparing Nrf2 and TGF-β1 protein levels in B16F10 cells with or without SB431540 (10 μM) treatment at 48 h. (**B**) Quantification of Nrf2 and TGF-β1 expression levels after treatment with or without SB431540, measured through Western blotting at 48 h. (**C**) Western blot analysis comparing Nrf2 and TGF-β1 in B16F10 cells treated with or without brusatol (40 nM) at 48 h. (**D**) Quantification of Nrf2 and TGF-β1 expression levels at 48 h after treatment with or without brusatol, as determined through Western blotting. Data are presented as the mean ± standard error of the mean after ≥3 independent experiments. Statistical significance was assessed using a two-tailed Student’s *t*-test: ** *p* < 0.01.

To determine whether Nrf2 also plays a role in TGF-β expression, we used SB431540, a TGF-β inhibitor, and brusatol to evaluate the expression of TGF-β in B16F10 cells containing HUVEC mitochondria. SB431540 inhibited TGF-β1 expression but did not affect Nrf2 expression (Figure 6A,B). By contrast, brusatol inhibited the expression of both Nrf2 and TGF-β1 (Figure 6C,D), indicating that Nrf2 plays a role in TGF-β expression in melanoma containing endothelial mitochondria.

## 3. Discussion

The TME plays a vital role in cancer development. Melanoma cells rely on their interactions with various other cells in their TME because such interactions are crucial for acquiring the characteristics typical of solid cancers. ECs are among the key interacting cell types in the gynecologic melanoma microenvironment. Studies have highlighted that although several mechanisms facilitate crosstalk between cancer and stromal cells, mitochondrial transfer supports cancer progression [22,35]. Thus, other studies have extensively investigated the effects of mitochondrial transfer on cell survival and antiapoptotic processes [24,35]. Cancer-associated fibroblasts (CAFs), which are predominant in the stromal compartment of many solid cancers, contribute to both tumor initiation and tumor progression. In particular, CAFs promote prostate cancer malignancy through mitochondrial transfer and metabolic reprogramming [36]. However, the role of endothelial mitochondria in melanoma progression remains unclear. Multiple methods for delivering exogenous mitochondria to recipient cells have been developed. Mitochondrial transplantation is one such method used to specifically examine the functional changes in recipient cancer cells after the uptake of mitochondria from donor cells in the TME. Various approaches—including coculturing, direct injection, and intracoronary vascular infusion in animal models—can be employed for mitochondrial transplantation [28]. Coculturing is the simplest approach for investigating the effects of mitochondrial uptake on the function of recipient cells. In addition, exogenous mitochondria can be introduced into cells through cell fusion, actin-dependent endocytosis, or micropinocytosis [37]. The present study investigated the effects of endothelial mitochondrial transplantation on melanoma progression.

Specifically, this study demonstrated that transplanting exogenous heterologous mitochondria from HUVECs into B16F10 melanoma cells resulted in the successful uptake of these mitochondria, thereby promoting the survival and proliferation of melanoma cells. However, the intricate mechanisms underlying this process remain unclear. Melanoma cells exhibit a high proliferative capacity mainly because of the constitutive activation of the ERK and AKT pathways, which results in rapid cell growth through the upregulation of cyclin D1 and cyclin E [38,39,40]. The increased expression of cyclin D1 in both primary and metastatic melanoma [41] indicates its crucial role in tumor progression. Although the caspase-3 increased and then decreased at 48 h, in the present study, after the transplantation of HUVEC mitochondria, B16F10 cells exhibited enhanced ERK and AKT signaling and increased cyclin D1 and cyclin E expression, suggesting that the transfer of endothelial mitochondria from the TME supports the growth of melanoma, a finding corroborated by relevant studies. Mitochondrial transfer between ECs and cancer cells causes phenotypic changes and induces chemoresistance in cancer cells [22]. In addition, one study demonstrated that stem cells donate their mitochondria to neighboring cells, aiding oxidative stress resistance and improving metabolic conditions, and thus promoting cell proliferation and enhancing antiapoptotic capability [42]. For instance, the introduction of healthy mitochondria into human prostate cancer PC-3 cells promoted cell proliferation and rescued cells from cisplatin-induced death [23]. Moreover, the engulfment of foreign somatic-derived mitochondria by MSCs increased the expression of the cytoprotective enzyme HO-1 and stimulated mitochondrial biogenesis [42].

HO-1 is a downstream protein of Nrf2 [30]. In this study, after the transplantation of HUVEC mitochondria, we observed increases in Nrf2, HO-1, PGC-1α, and Sirt1 levels. Sirt1 plays a key role in metabolic control and regulates the proliferation and viability of melanoma cancer cells [43]. It also contributes to mitochondrial biogenesis and maintains cellular redox homeostasis by increasing PGC-1α expression [44] and promoting Nrf2 activation [45]. Nrf2 activation promotes the transcription of cytoprotective genes and antioxidant enzymes, thereby protecting against oxidative stress in melanoma [46]. Our findings revealed that the autophagic marker LC3B was downregulated in melanoma cells and in a xenograft tumor animal model. Autophagy modulation occurs through an ROS-dependent mechanism. Inhibition of the antioxidant protein HO-1 induced autophagy in cancer cells [47]. As depicted in Figure 3E, the ROS level was reduced by the Nrf2 inhibitor. These results suggest that autophagy is inhibited through Nrf2/HO-1-mediated ROS elimination in melanoma cells containing endothelial mitochondria. In addition, AKT phosphorylation activates Nrf2-dependent mitochondrial biogenesis [48]. In the present study, the Nrf2 inhibitor suppressed AKT signaling (Figure 3C), suggesting that Nrf2 regulates AKT activity in melanoma cells through a positive feedback loop after the uptake of endothelial mitochondria. TGF-β promotes MMP-9-mediated tumor invasion [49]. In addition, TGF-β1not only promotes cancer cell invasion and metastasis, but also induces M2-type TAM polarization. Macrophages in primary malignant melanoma may contain the melanin pigment [50]. M2-type TAMs are recognized as a predictor of poor prognosis in patients with cutaneous malignant melanoma [51]. Finally, brusatol suppressed TGF-β (Figure 6C), indicating that Nrf2 may play a role in TGF-β1-induced M2-type TAM polarization after the uptake of mitochondria from ECs in the TME of melanoma cells.

**Figure 7:**
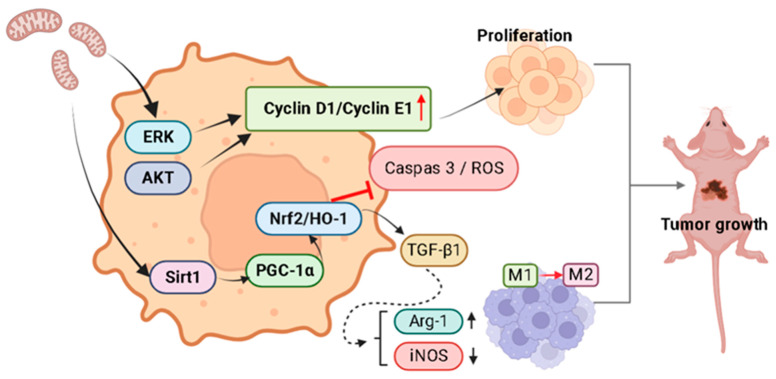
Schematic depicting how exogenous mitochondria from ECs can regulate melanoma tumor growth by activating proliferative signaling pathways, upregulating antioxidant molecules, and subsequently inhibiting apoptosis. Exogenous endothelial mitochondria activate the ERK/AKT and Sirt1/PGC-1a/Nrf2/HO-1 signaling pathways in melanoma cancer cells. The activated ERK/AKT signaling induces cyclin D1/cyclin E1-mediated cell proliferation. Upregulation of Sirt1/PGC-1a/Nrf2/HO-1 signaling inhibits ROS and caspase-3 activation and induces M2 macrophage polarization (by Arg-1 upregulation and iNOS downregulation) by Nrf-2/TGF-β signaling, thereby promoting tumor growth.

The present study observed a time-dependent increase in AKT phosphorylation following mitochondrial transplantation, suggesting that endothelial mitochondrial transplantation contributes to melanoma proliferation by enabling AKT-mediated PGC-1α–Nrf2-dependent mitochondrial biogenesis and cellular redox homeostasis, which in turn promotes cell proliferation and inhibit apoptosis. Nrf2 was involved in endothelial mitochondria transfer-mediated melanoma growth (Figure S1). Moreover, Nrf2 plays a role in TGF-β1-induced M2-type TAM polarization after the uptake of mitochondria from ECs in the TME of melanoma cells. Xenograft experiments revealed that tumors in the experimental group were significantly larger than those in the control group. These results indicated that melanoma cells became more proliferative following the incorporation of exogenous mitochondria into their mitochondrial network (Figure 7). This finding further supports the role of the endothelium in tumor development in the TME.

## 4. Materials and Methods

### 4.1. Cell Culture and Reagents

B16F10 melanoma cells, purchased from Merck (Darmstadt, Germany), were cultured in Dulbecco’s Modified Eagle’s Medium (DMEM) (Gibco, Waltham, MA, USA) supplemented with 10% FBS. Human umbilical vein ECs (HUVECs), purchased from Thermo Fisher Scientific (Waltham, MA, USA), were cultured in the M199 medium supplemented with 20% fetal bovine serum (FBS). Mouse monocyte macrophage RAW264.7, purchased from ATCC (Manassas, VA, USA), were cultured in DMEM (Gibco, Waltham, MA, USA) supplemented with 10% FBS. These cells were incubated at 37 °C in a growth chamber containing CO_2_ (5%). Brusatol (MedChemExpress, NJ, USA), AI-1 (Focus biomolecules, Plymouth Meeting, PA, USA), and SB431542 (Sigma-Aldrich, St. Louis, MO, USA) in dimethyl sulfoxide (DMSO) (Sigma-Aldrich, St. Louis, MO, USA) were prepared and dissolved in a culture medium before treatment. Recombinant Human TGF-β1 (Cell Guidance Systems; GFH39-5, Cambridge, UK) was prepared and dissolved in a culture medium.

### 4.2. Isolation of Mitochondria and Mitochondrial Transplantation

Mitochondria were isolated following the protocol provided by Abcam. The cells were subjected to trypsinization and then collected through centrifugation. Lysis was performed using 500 μL of cytosol extraction buffer followed by moderate shaking for 20 min. The mixture was then centrifuged at 700× *g* for 20 min, after which the supernatant was transferred to a new tube. Additional centrifugation at 10,000× *g* was performed to precipitate the mitochondria. The mitochondria isolated from 5 × 10^6^ HUVECs were co-incubated with B16F10 cells for a specified duration.

### 4.3. Cell Viability Analysis

B16F10 melanoma cells were seeded in 96-well dishes in quadruplicate at 6000 cells/well and cultured for 24 h before mitochondrial transplantation. Cell viability was analyzed using Cell Counting Kit-8 (Sigma–Aldrich, St. Louis, MO, USA) and absorbance was measured at 450 nm by using a microplate reader.

### 4.4. Cellular ROS Assay

The cells were harvested and washed with phosphate-buffered saline (PBS) and stained with 2',7'-dichlorodihydrofluorescein diacetate (H_2_DCFDA; Med Chem Express, Monmouth Junction, NJ, USA) for 15 min. The cells were then washed twice with cold PBS, and analyzed through flow cytometry.

### 4.5. Caspase Activity Analysis

The cells were harvested, washed with PBS, and stained with a Cleaved Caspase-3 Staining Kit (Abcam, Cambridge, UK). The stained cells were then analyzed through flow cytometry.

### 4.6. Western Blot

Total proteins from the B16F10 cells into which HUVEC mitochondria were transplanted were extracted using a lysis buffer composed of HEPES (50 mM, pH 7.7), EDTA (1 mM), neocuproine (0.1 mM), and CHAPS (0.4%, *w*/*v*). Forty micrograms of proteins were mixed with a sample buffer containing Tris-HCl (62.5 mM, pH 6.8), SDS (3%, *w*/*v*), 2-mercaptoethanol (5%, *v*/*v*), and glycerol (10%, *v*/*v*). The proteins were then separated through SDS–polyacrylamide gel electrophoresis and transferred to a PVDF membrane (Millipore, Billerica, MA, USA). The membrane was incubated with primary antibodies against Nrf2 (1:1000; ABclonal, Woburn, MA, USA), PGC-1α (1:1000; NOVUS, CO, USA), sirt1 (1:1000; ABclonal, Woburn, MA, USA), HO-1 (1:1000; Cell Signaling, Danvers, MA, USA), LC3B (1:1000; Cell Signaling, Danvers, MA, USA), AKT (1:1000; Cell Signaling, Danvers, MA, USA), p-AKT (1:1000; Cell Signaling, Danvers, MA, USA), ERK (1:1000; Cell Signaling, Danvers, MA, USA), p-ERK (1:1000; Cell Signaling, Danvers, MA, USA), cyclin D1 (1:1000; Cell Signaling, Danvers, MA, USA), Cyclin E (1:1000; Cell Signaling, Danvers, MA, USA), caspase-3 (1:1000; Cell Signaling, Danvers, MA, USA), cleaved caspase-3 (c-caspase-3) (1:1000; Cell Signaling, Danvers, MA, USA), MMP9 (1:1000; arigo Biolaboratories, Hsinchu City, Taiwan), TGF-β1 (1:1000; Abcam, Cambridge, UK), iNOS (1:1000; ABclonal, Woburn, MA, USA), Arg-1 (1:1000; Proteintech, Planegg-Martinsried, Germany), or β-actin (1:1000; Cell Signaling, Danvers, MA, USA) at 4 °C. Subsequently, the membranes were incubated with secondary antibodies at room temperature for 1 h and analyzed using an electrochemiluminescence detection system.

### 4.7. Xenograft Tumor Experiments

After the HUVEC endothelial mitochondria had been transplanted into the B16F10 cells, these cells were then harvested and subcutaneously inoculated (5 × 10^6^ cells/0.1 mL in PBS) into 6-week-old BALB/c nude mice (BioLASCO, Taipei City, Taiwan). The tumor volume was calculated using the formula V = L × W^2^/2 (L, length; W, width) and the tumors were harvested after 10 days.

### 4.8. Statistical Analysis

All data were analyzed using GraphPad Prism version 8 (GraphPad Software, San Diego, CA, USA). Results are presented as the mean ± standard error of the mean. Statistical significance was determined using Student’s *t*-test, with the significance threshold set at *p* < 0.05.

## 5. Conclusions

The present study demonstrates that exogenous mitochondria from ECs can regulate the growth of melanoma tumors. This regulatory effect is achieved through the activation of proliferative signaling pathways, the upregulation of antioxidant molecules, and the subsequent inhibition of apoptosis. These results provide valuable insights into the role of mitochondrial transfer from ECs to melanoma cells in the regulation of the TME, and highlight potential targets for the treatment of gynecologic tract melanoma.

## Data Availability

All data sets generated or analyzed in this study are included in the published article. Detailed data sets supporting the current study are available from the co-responding author upon request. This study did not generate new codes.

## Supplementary Materials

The following supporting information can be downloaded at: https://www.mdpi.com/article/10.3390/ijms25031857/s1.
