# Supplementary material for: Endothelial Mitochondria Transfer to Melanoma Induces M2-Type Macrophage Polarization and Promotes Tumor Growth by the Nrf2/HO-1-Mediated Pathway
Source: Int J Mol Sci. 2024 Feb 3;25(3):1857. doi: 10.3390/ijms25031857 (PMC10855867; doi:10.3390/ijms25031857)
Supplement: Supplementary file 1 [file ijms-25-01857-s001.zip › ijms-2823219-supplementary.pdf]

Supplementary figure

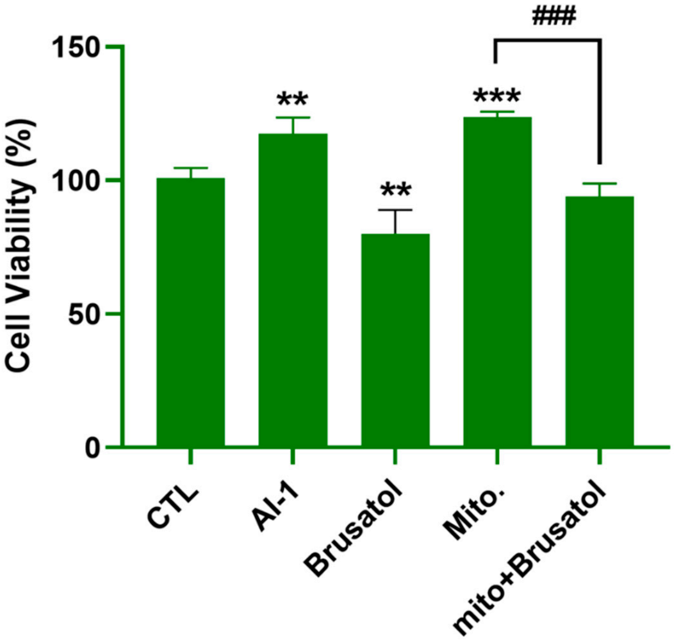

Figure S1. Viability of B16F10 cells at 48 h after treatment (AI-1: Nrf2 activator; brusatol: Nrf2 inhibitor; Mito: endothelial mitochondria), as evaluated using the CCK8 assay. The AI-1 (10 μM; Nrf2 activator) or brusatol (40 nM; Nrf2 inhibitor) was used to evaluate cell viability at 48 h by CCK-8. The results demonstrated that Nrf2 activator increased cell viability, however, Nrf2 inhibitor suppressed cell growth. Nrf2 inhibitor, brusatol, significantly reversed endothelial mitochondria-induced cell growth, suggesting that Nrf2 was involved in endothelial mitochondria transfer-mediated melanoma growth. Statistical analysis was used to determine if treatments were not significant, or significant compared to control (CTL) at P<0.01 (**) or P<0.005 (***) or significant compared to Mito group at P<0.005 ($^{\#\#\#}$). Analysis was performed on five independent experiments and the mean values (±SEM) shown.
